# Aligning provider incentives to improve primary healthcare delivery in the United States

**DOI:** 10.13172/2052-8922-1-1-958

**Published:** 2013-06-01

**Authors:** JE DeVoe, R Stenger

**Affiliations:** 1 Department of Family Medicine, Oregon Health and Science University, 3181 Sam Jackson Park Road, Mailcode: FM, Portland, OR 97239, USA; 2 Saint Patrick Hospital, 500 West Broadway Street, Missoula, MT 59802, USA

## Abstract

**Background:**

The United States (US) is reforming primary care delivery systems, including the implementation of ‘patient-centered medical homes.’ Alignment of provider incentives with desired outcomes will likely be important to the success of these delivery system reforms.

**Methods:**

This critical review uses a theoretical framework from game-theory models to discuss some of the dominant primary care provider payment models and how they create ‘prisoner’s dilemmas’ that have stalled past reform efforts. It then uses this framework to illustrate, hypothetically, how advantages from different models could be blended together to encourage cooperation and improve the quality of primary care services delivered, thus providing an escape from current prisoner’s dilemmas faced by providers.

**Findings:**

Improvements in primary care delivery will largely hinge on blended payment mechanisms that can effectively combine the advantageous elements of fee-for-service, capitation, and incentive payments into a balanced equation that enables providers to escape the perverse financial incentives of current payment mechanisms and overcome collective action problems.

**Conclusions:**

If balanced appropriately, a blend of guaranteed payment and selective incentives designed to encourage primary care providers to deliver high quality care, efficient and equitable care and to eliminate incentives towards over-servicing could reach outcomes leading to shared benefits for everyone involved.

## Introduction

### Background

As the United States (US) grapples with fixing its broken health care system, reforming the primary care delivery system and implementing the ‘patient-centered medical home’ model have featured prominently in recent debates.^1,2,3,4,5,6^.

The 2010 Patient Protection and Affordable Care Act (the ‘Obama Health Care Plan’) included specific provisions for expanding new models of primary care.^7^

There is increasing awareness that changing the US primary care delivery system will also require changing payment and reimbursement models to better support patient-centered, comprehensive, coordinated and continuing care. Many experts have called for movement away from fee-for-service towards capitation or a blended payment approach.^8,9,10,11,12^ However, there is currently limited information and serious skepticism about how (and if) a blended mechanism could work effectively.^2, 13,14,15,16^ In this critical review, we use the ‘prisoner’s dilemma’ aspect of economic game theory to highlight perversities inherent in current primary care payment mechanisms and to illuminate how blended payments might provide an escape from perverse financial incentives that prevent primary care providers from reforming the health care system to better promote health.^17^

### Definitions

The term ‘**capitation**’ has come to describe several aspects of the organization, management, delivery, and financing of health care services. Proponents of capitation argue that it creates incentives for population health management, efficiency and cost-containment while, at the same time, increasing the quality of services^18,19, 20,21^. Opponents warn that the underlying incentives lead to under-servicing and the provision of inadequate care.^22,23^ Capitation has been linked to public fears about the quality of care,^22,24,25,26^ dissatisfaction among providers,^27,28,29^ and the erosion of patient trust in medical professionals.^30^

For the purposes of this paper, capitation refers only to a mechanism of payment. Capitation involves arranging a global fee to be paid in advance to a provider (an individual, a group of providers, or an institution employing providers delivering primary care services) in return for the ongoing care of a patient over a specified period of time and across a defined scope of services. An exhaustive discussion of capitation formulas is beyond the scope of this paper, and can be found elsewhere.^31,32,33,34,35,36,37,38^

However, it is important to mention that most methods for calculating capitation rates attempt to risk adjust by taking into account the past history of diagnoses and service provision among a collective group of patients and providers. Regardless of the method used to estimate rates, a capitation contract transfers risk to providers. A provider stands to gain if the rate is higher than the level of service provided. Alternatively, a low rate will result in a financial loss for the provider. Further, there has been considerable debate about whether risk adjustment methodologies adequately predict the level of service needed by specific patients or populations.^22,23, 31,32,33,34,35,36,37^

At the other end of the spectrum is **fee-for-service**, which pays a provider for each service. There are many formulas for determining fees, and procedural services are usually valued much higher than cognitive services. Fee-for-service has been criticized for providing incentives towards over-servicing.^39,40,41^

Fee-for-service models differ from capitation (simplistically) because providers are guaranteed a return on every effort expended, and thus have direct incentives to provide services that yield the highest payment, regardless of supporting evidence. If services are not required, providers do not get paid. Alternatively, providers benefit if the need (or desire) for services is abundant.^42^

In pure capitation and fee-for-service models, providers are relied upon to determine what services are necessary, and these same providers face a financial gain or loss from the provision of these services.^43^ In each of these pure payment models, providers face financial incentives that create a ‘dilemma,’ which juxtaposes maximizing financial reimbursement versus providing the right amount of services. To achieve optimal population health and an economically viable and sustainable health care system, it will be essential to create new models of provider payment that enable an escape from these dilemmas and foster ‘cooperation’ among providers. We have coined the term ‘cooperative jailbreak’ to represent an escape from the prisoner’s dilemmas inherent in the most common provider reimbursement models.

### Methods

In this section, we use aspects of the prisoner’s dilemma model to frame discussions about the perverse incentives of current primary care provider reimbursement mechanisms and to illustrate, in theory, the potential for meaningful payment reform that could enable a focus on health.^17^ We acknowledge the extent to which we have simplified assumptions in these models.

#### Game Theory and the Prisoner’s Dilemma Model

Game theory has been used to predict the actions of individuals in strategic situations (or ‘games’) where the success of one individual depends on the actions of others.^44,45^ The prisoner’s dilemma describes a particular problem in a non-zero sum game where a ‘player’ faces a choice to ‘cooperate’ with other players or to ‘defect.’ The resulting possible scenarios are classically represented in a 2×2 table (Table 1). The highest payoff for a player is to ‘defect’ while the other players cooperate (BOX#3, Player 1). In this scenario, if only one player cooperates while the others defect, he or she receives the lowest payoff (BOX#2, Player 1). If all players cooperate, they each get the second highest payoff (Box #1), and if all defect, they each receive the second lowest payoff (Box #4).

If only one cycle of the game is played, defection is the dominant strategy. This strategy is the only way a player is assured at least 1 point and has a chance to achieve 5 points. An assignment of strategies is termed a Nash equilibrium if no player could have improved his or her pay-off by deviating from the assigned strategy, given that all other players also play their assigned strategies.

For example, if all players defect (Table 1, Box #4), no individual player could have improved his or her payoff by choosing to cooperate. If the game is repeated, however, there is potential for system-wide improvement if all players cooperate as indicated in Table 1, Box #1 which yields the most total points = 6 and an increase in the individual gain (3 points each). Individuals are faced with a ‘prisoner’s dilemma’ in deciding to either cooperate and hope the other player also cooperates (receiving the 2^nd^ highest individual and the highest overall system pay-off) or to defect and hope the other player cooperates, thus earning the maximum individual payoff of 5 points. (This defection by only one player while all others cooperate is referred to as ‘free riding,’ discussed further below.)

#### Adaptation of the ‘Prisoner’s Dilemma’ Model in Incentives in Primary Care

The game-theoretic models illustrate the payoff for an individual provider, which depends on his or her own behavior and the behavior of all other providers. It is assumed that each provider’s payment depends on three main components: financial remuneration, costs, and externalities, which are represented by five variables:

**V** - Volume of services
**Q** - Quality of care
**C** - Capitation rate
**E** – Externalities (i.e., benefits or costs that accrue to individual providers as a result of the overall environment)
**I** - Incentive payments - payments to individual providers for specific outcomes (e.g., meeting quality benchmarks, coordination of care).

The relevance of each variable to a provider’s payoff depends, in large part, on the underlying payment mechanism (e.g., fee-for-service, capitation, blended payments). To a certain extent, it also depends on externalities, which represent the benefits or burdens accruing to a provider by the overall performance of the system of interconnected providers (e.g., a better than average performance of the medical system might improve health outcomes and, therefore, reduce future costs to all providers). Externalities are a result of the overall environment as determined by the collective action of most providers (e.g., standard of care, improvement of overall community health status).

These game theoretic models are also built on the assumption that ultimate provider payment is based on the providers’ decisions about the volume and quality of service to provide. For simplicity, we assume providers can control the volume (***V*_high_** or ***V*_low_**) and the quality of care (***Q*_high_** or ***Q*_low_**) provided. We also assume that the externalities that accrue to individual providers depend primarily on the quality of care provided by most providers (if **Q**=***Q*_high_** then **E**=***E*_highQ_**). While the assumption of binary variables simplifies our models, a continuous representation of possible levels of service and quality would support similar conclusions. Table 1 depicts two ‘players’ in the game. In health care, Player 2 represents the collective group of ‘others’ (usually more than one individual). The number of providers represented by Player 2 would likely influence the strategy choice (i.e., the more players involved, the more likely that defection of any one of them will occur). This potential for variable influence based on player scale is incorporated into the externality concept (E).

## Results

### The Fee-for-Service Model

Under the fee-for-service model, a provider’s payment depends primarily on volume of services with little or no incentive to address quality, efficiency or equity in the provision of health care. There are no capitation or incentive payments. In addition, we assume that externalities resulting from an overall high quality of care could slightly reduce an individual provider’s payoff by improving the health of the population and decreasing the need for more services. We present only a model of high volume, assuming that few or no providers would choose to provide a lower volume of service (which would yield a lower payment). Further, providers choosing to increase the quality of care in ways that do not generate a fee incur additional costs for which they do not receive compensation. Using these assumptions, we assigned the following values for each variable: V_high_=5, V_low_=3; C=0; E_highQ_=−1, E_lowQ_=0; Q_high_=−2, Q_low_=0; I=0. Thus, under a fee-for-service payment model, provider payment (PP) is represented by the formula: ***PP = V+Q+E***. The four possible outcomes are represented in Table 2.

Regardless of what other providers do, providing the highest number of low quality services is the dominant economic strategy for all agents. In game-theoretic terms, this becomes the (only) Nash equilibrium. This is consistent with the standard view that fee-for-service incentivizes over-servicing (charging the highest possible price to the patient and to the system). Under this model, there is no prisoner’s dilemma as players have no incentive to cooperate with each other to equitably distribute services that maximize population health, as these efforts will likely diminish the individual economic payoff. Only in cases where maximization of individual payoff threatens the integrity of the overall system (i.e., high rates of uninsured individuals) would providers consider changing their dominant strategy.

This representation of fee-for-service assumes providers would always choose quantity over quality to achieve maximum gain: low quality services are cheaper than high quality services, and thus would be the dominant economic strategy. This is the theoretical dominant strategy; however, in reality, we recognize that providers choices would vary based on multiple additional factors (e.g., desire to build good relationships with patients, altruism, etc). These non-economic influences, however, would serve to reduce maximum payoff.

### The Capitation Model with a Short Time Horizon

A capitation model with a short time horizon also yields one dominant strategy. In this model, a provider’s payoff consists of a global capitation rate minus the cost (to the provider) of delivering care. We assume that most providers would choose to provide a lower volume of services, and providers that choose to increase the quality of care incur additional costs for which they do not receive compensation. There are no incentive payments, and we assume that externalities may minimally reduce providers’ costs if all providers deliver high quality care (and thus fewer patients may need a high volume of services). Using these assumptions, we assigned the following values for each variable: V_high_= −2, V_low_ =0; Q_high_=−2, Q_low_=0; C= 5; E_highQ_=1, E_lowQ_=0; I=0. Thus, under a capitation model provider payment is represented by the formula ***PP = V+Q+C+E*** and the four possible outcomes are represented in Table 3.

Again, regardless of what all other providers choose to do, the dominant strategy is to provide a low number of low quality services (***V*** always = 0). Thus, low volume/low quality is, in game-theoretic terms, the (only) Nash equilibrium. This observation is consistent with the standard view that a pure capitation model sets a financial incentive to provide the lowest number and the lowest quality of services (and hope that other providers provide more adequate levels of service to maximize the externalities), regardless of the size of the capitation rate.

This representation of capitation in game theoretic terms does assume providers would always choose the lowest quantity and lowest quality to achieve maximum gain, which is defined in a narrow economic sense here. In reality, we recognize that providers’ choices would vary based on multiple additional factors, which would serve to reduce maximum financial payoff but would have other non-financial benefits.

### The Capitation Model with a Longer Time Horizon – (A Prisoner’s Dilemma)

If future capitation rates are based on prior costs, the dominant strategy in Table 3 has the potential to backfire over a longer time horizon. If the collective group of providers repeatedly delivers a low volume of low quality services, the global capitation rate for future periods is likely to decrease (as will the health of the population requiring the need for a higher volume of services in the future). Table 4 shows the payoff matrix for a future period of time when the capitation rate has either remained high based on prior high overall quality or decreased significantly based on prior low overall quality (***C***= either ***C***_highQ_=5 or ***C***_lowQ_=3). We assume the other variables retain the same values as in the previous short-term capitation example.

Once more, the (unique) Nash equilibrium is to deliver a low volume of low quality services. But this time, the situation is a prisoner’s dilemma, because the overall system payoff (and individual payoff in the long term) could be increased if all providers cooperated to deliver high quality care. Each provider is faced with a collective action problem of the kind famously identified by Mancur Olson^44^: future payoff depends on the externalities of the average quality of services, which depends on the present choices of all providers. But each provider has an incentive to free-ride, which discourages collective action. If most other providers offer high quality service (costing them more per service), an individual provider benefits from being in this environment with an adequate supply of high quality services (or by ‘cherry-picking’ the most healthy patients), irrespective of this provider’s contribution.^46, 47^ And, if most others choose the dominant strategy and continue to provide low quality services, an individual provider is adversely affected by being the only one who makes the necessary investments to provide high quality services (or to provide care for the most vulnerable patients with the highest co-morbidities).

The prisoner’s dilemma lies in the fact that individually rational behavior (defined in the game-theoretic terms as pursuing the highest individual payoff) leads to a collectively sub-optimal outcome. In order to achieve the collectively superior outcome, or a ‘cooperative jailbreak’ from the prisoner’s dilemma, a cooperative disposition against the incentive to free-ride would be required on the part of every provider. This could be accomplished by adding appropriate incentives into the system to encourage providers to ‘do the right thing.’ In addition, the rules governing the system must allow for cooperation amongst providers.^48, 49^

### Blended Payments – A Cooperative Jailbreak That Decreases the Potential to Free-Ride?

Could blended payment mechanisms help providers escape the urge to free-ride? Could blended payments incentivize high quality care delivered efficiently and equitably while also ensuring the appropriate use of evidence-based information?^50–53^ Several experts have contributed to theoretical discussions about how to solve the prisoner’s dilemma and other related collective action problems.^45, 54, 55^ One solution involves changing the incentive structure, so as to remove the prisoner’s dilemma. Specifically, in addition to the collective benefits from maximizing quality, the idea is to create benefits that accrue selectively for each provider on the basis of whether he or she chooses to provide high or low quality care and the appropriate volume of services.

The predominant blended payment models involve three components: (1) fee-for-service payments for some services, (2) a prospective care-management capitation fee, and (3) incentive payments based on achieving some desirable outcomes (e.g., achieving benchmarks, meaningful use of an electronic medical record).^8, 56^ In such a system, a provider’s base payment could be dominated either by the fee-for-service or capitation portions, and the additional incentive payments could be either large or small relative to the primary source of payment. In this situation, provider payment is calculated as follows: ***PP= V+Q+C+E+I*** where incentive payments (***I***) would be dependent on an individual provider’s quality of care in some prior period.

Table 5 demonstrates the impact of adding a small or a big incentive payment to a model that is predominantly based on fee-for-service or capitation. To reach a maximum payment =6, we first assumed that a small incentive payment (***I***=1) would be a small percentage (e.g., 20%) of a ‘base’ fee-for-service+capitation payment of 5. When the quality of services is high, then ***I***_highQ_=1; when quality is low, then ***I***_lowQ_=0. Or, alternatively, assume that a bigger incentive payment (I=2) would be a larger percentage (e.g., 50%) of a slightly lower ‘base’ payment of 4. In this example, when the quality of services is high then ***I***_highQ_=2; when quality is low, then ***I***_lowQ_=0. We also assume that the addition of any fee-for-service payment will encourage a higher volume of services.

Tables 5a and 5b include a small incentive payment; the Nash equilibrium is for all providers to provide a high volume of low quality services. There is also the temptation to free ride by providing low quality services in a high quality environment. In Tables 5c and 5d, which includes a slightly lower base and bigger incentive payment, however, there is no Nash equilibrium and no incentive to free ride. Thus, an individual provider could potentially be motivated to choose the strategy of high volume/high quality that provides the greatest collective benefit. If future capitation rates in examples c and d were risk-adjusted based on the delivery of current high quality care (as in table 4), the incentive for providers to deliver high quality care could potentially be magnified.

## Discussion

Payment models are central to health care delivery and will be key to improving the primary care delivery system. There is a need to investigate how different incentive structures could lead to the delivery of high quality care, efficiency and equity.^17^ Game theoretic models are useful to heuristically highlight how different payment models might work in real-world settings. Whether or not, and to what extent, providers actually face collective action problems depends on many empirical specifics. For example, for a longer-term capitation model to create a prisoner’s dilemma: (1) future capitation rates must closely track the previous average level of service, and (2) positive externalities of a high average quality of care must affect individual providers.

If both criteria hold true, to some extent, providers face a serious prisoner’s dilemma that could potentially be made better or worse by introducing alternative payment methodologies into new primary care delivery models.^3, 57^ One such alternative is a blended payment mechanism that effectively combines the elements of fee-for-service, capitation, and incentive payments.^50, 51,52,58,59^ Blended payments have the potential to tip the incentive structure towards cooperation, resulting in improved health care delivery, maximizing population health and minimizing costs—achieving the ‘triple aim.’^60^

But, the applicability of these models also depends on the ability of providers to effectively collaborate and to overcome many of the obstacles that have made it difficult to build synergistic partnerships in the past.^48,61,62,63,64,65^

The inclusion of a pay-for-performance component (e.g., fee-for-service) in blended payment models also warrants some discussion. For the purposes of our models, we assume that this component would encourage providers to deliver a higher volume of services, which has been shown in recent empiric studies.^66,67^ To the extent that care delivered by a primary care provider is relatively efficient compared to similar care delivered in other settings,^68,69,70,71,72^ we believe it may be necessary to retain a mechanism that encourages primary care providers to offer a higher volume of appropriate services themselves, rather than refer cases out.^73^

### Special Considerations and Next Step

The capitation model that extends over a longer time horizon is still finite and thus cannot account for special circumstances, such as an individual solo practitioner nearing retirement who may revert back to the dominant behavior of those providers with only a short horizon.

We also acknowledge that individual providers are rarely isolated or independent; rather, they are part of a highly interconnected network of providers, and the behavior of each provider (and multiple third parties) will affect the others. Generally, the relevance of game theory models depends on how strongly the effects of different providers’ actions are interconnected. Further, the type of provider (e.g., solo practice, small group practice, large group network) being reimbursed greatly affects the model, and complex systems might contain different levels of payment (i.e., an individual provider may have a different incentive system than the larger entity). Regardless of size and underlying structure, however, all providers ultimately do face decisions regarding the volume of services to provide and the quality of those services.

The purpose of this paper is not to provide a comprehensive description of the highly complex economic mechanisms and incentive structures in health care systems. We recognize that more sophisticated frameworks are needed to develop and refine blended payment models that achieve true payment reform and optimally improve health care delivery that better achieves health.

We propose that case studies describing empiric tests of how these theoretical models could apply to and be modified by ‘real world’ settings and demonstrations of practice transformation are logical next steps.

## Conclusions

We highlight a novel theoretical framework to review predominant payment models and to inform efforts to align payment incentives to improve primary health care delivery. Both empirical investigations (i.e., current pilot or demonstration projects) and detailed modeling of blended payment schemes are needed to determine how to design incentives that will make the delivery of high quality care the dominant strategy for every health care provider and system. If successful, the right combinations of ethical incentives built upon sound form of blended payment could provide a cooperative jailbreak from prisoner’s dilemmas and ultimately lead towards an integration of care and innovation in system design.^22,39,58^

The rapid adoption of new health care delivery models and provider payment schemes, despite little empirical evidence, has created daunting challenges for researchers: How might experiments in provider payment within these new demonstration projects impact the current health care system?^3^ More pressing, is it even possible to reach the goal of a payment system that promotes both increased quality and reduced costs?^13, 50^ This paper was intended to shed some optimistic light on how we might meet this challenge with regards to the reimbursement of primary care providers. We hope that our illustration of the theoretic potential of blended payments will encourage further research and development of blended payment models.

## Figures and Tables

**Table: 1:** The Prisoner’s Dilemma Model.

	**Player 2 (“others”):** **COOPERATE**	**Player 2 (“others”):** **DEFECT**
**Player 1:** **COOPERATE**	Player 1 = 3 points Player 2 = 3 points = 2^nd^ highest payoff for Player 1 = 2^nd^ highest payoff for Player 2 (BOX #1)	Player 1 = 0 points Player 2 = 5 points = lowest payoff for Player 1 = highest payoff for Player 2 (BOX #2)
**Player 1:** **DEFECT**	Player 1 = 5 points Player 2 = 0 points = highest payoff for Player 1 = lowest payoff for Player 2 (BOX #3)	Player 1 = 1 point Player 2=1 point = 2^nd^ lowest payoff for Player 1 = 2^nd^ lowest payoff for Player 2 (BOX #4)

**Table: 2:** The Fee-For-Service Model.

Provider Payment (PP) = Volume of Services (V) + Quality of Care (Q) + Externalities (E) Using these assumptions: V_high_=5, V_low_=3, E_highQ_ =−1, E_lowQ_ =0, Q_high_=−2, Q_low_=0. (no capitation rate, no incentive payment)		
	**Provider 2 (P2)** **(“others”)** **(high volume, low quality)**	**Provider 2 (P2)** **(“others”)** **(high volume, high quality)**
**Provider 1 (P1)** **(high volume,** **low quality)**	P1 = 5 (5+0+0) P2 = 5 (5+0+0)	P1 = 4 (5+0−1) P2 = 2 (5−2−1)
**Provider 1 (P1)** **(high volume,** **high quality)**	P1 = 3 (5−2+0) P2 = 5 (5+0+0)	P1 = 2 (5−2−1) P2 = 2 (5−2−1)
**Results**: the dominant strategy is for all players to provide a high volume of low quality services.		

**Table 3:** The Capitation Model with a Short Time Horizon.

Provider Payment (PP) = Volume of Services (V) + Quality of Care (Q) + Capitation Rate (C) + Externalities (E) Using these assumptions: V_high_= −2, V_low_ =0; Q_high_=−2, Q_low_=0; C= 5; E_highQ_=1, E_lowQ_=0 (no incentive payments)		
	Provider 2 (P2) (“others”) (low volume, low quality)	Provider 2 (P2) (“others”) (low volume, high quality)
Provider 1 (P1) (low volume, low quality)	P1 = 5 (0+0+5+0) P2 = 5 (0+0+5+0)	P1 = 6 (0+0+5+1) P2 = 4 (0+ −2+5+1)
**Provider 1** **(P1)** **(low volume,** **high quality)**	P1 = 3 (0+ −2+5+0) P2 = 5 (0+0+5+0)	P1 = 4 (0+ −2+5+1) P2 = 4 (0+ −2+5+1)
**Results** the dominant strategy is to pursue the highest individual payoff and provide a low volume of low quality services. There is an individual temptation to free ride by providing low quality services in a high quality environment [*i.e.,* if others are providing high quality services, then the exerternalities (E) = 1].		

**Table: 4:** The Capitation Model with a Longer Time Horizon.

Provider Payment (PP) = Volume of Services (V) + Quality of Care (Q) + Capitation Rate (C) + Externalities (E) Using these assumptions: V_high_= −2, V_low_ =0; Q_high_=−2, Q_low_=0; C_highQ_= 5, C_lowQ_ = 3; E_highQ_=1, E_lowQ_=0 (no incentive payments)		
	Provider 2 (P2) (“others”) (low volume, low quality)	Provider 2 (P2) (“others”) (low volume, high quality)
**Provider 1 (P1)** **(low volume,** **low quality)**	P1 = 3 (0+0+3+0) P2 = 3 (0+0+3+0)	P1 = 6 (0+0+5+1) P2 = 4 (0+ −2+5+1)
**Provider 1 (P1)** **(low volume,** **high quality)**	P1 = 1 (0+ −2+3+0) P2 = 3 (0+0+3+0)	P1 = 4 (0+ −2+5+1) P2 = 4 (0+ −2+5+1)
**Results** prisoner’s dilemma – the dominant individual strategy is to provide a low volume of low quality services. There is the potential for individual and system improvement with collective action to provide high quality services (which keeps the capitation rate = 5), but also a temptation for individuals to free ride in this scenario [*i.e.,* capitation rate stays at 5 if enough others are providing high quality services, and the externalities (E) = 1].		

**Table 5:** Blended Payment Examples.

Provider Payment (PP) = Volume of Services (V) + Quality of Care (Q) + Capitation Rate (C) + Externalities (E) + Incentive Payments (I)		
**5a**. Fee-For-Service Dominant Blended Payment with Small Incentive Payment **Using these assumptions: V_high_= 4, V_low_=0; Q_high_=−2, Q_low_=0; C= 1; E_highQ_=1, E_lowQ_=0; I_highQ_=1, I_lowQ_=0** **Most emphasis on paying for volume of services (V_high_= 4), with small capitation rate (C= 1) and small** **incentive payment (I_highQ_=1)**		
	**Provider 2 (P2) (“others”)** **(high volume, low quality)**	**Provider 2 (P2) (“others”)** **(high volume, high quality)**
**Provider 1 (P1)** **(high volume,** **low quality)**	P1 = 5 (4+0+1+0+0) P2 = 5 (4+0+1+0+0)	P1 = 6 (4+0+1+1+0) P2 = 5 (4−2+1+1+1)
**Provider 1 (P1)** **(high volume,** **high quality)**	P1 = 4 (4−2+1+0+1) P2 = 5 (4−0+1+0+0)	P1 = 5 (4−2+1+1+1) P2 = 5 (4−2+1+1+1)
**5b**. Capitation Dominant Blended Payment with Small Incentive Payment **Using these assumptions: V_high_= 1, V_low_ =0; Q_high_=−2, Q_low_=0; C= 4; E_highQ_=1, E_lowQ_=0; I_highQ_=1, I_lowQ_=0** **Most emphasis on capitation rate (C= 4), with small fee for volume of services (V_high_= 1), small incentive** **payment (I_highQ_=1)**		
	**Provider 2 (P2) (“others”)** **(high volume, low quality)**	**Provider 2 (P2) (“others”)** **(high volume, high quality)**
**Provider 1 (P1)** **(high volume,** **low quality)**	P1 = 5 (1+0+4+0+0) P2 = 5 (1+0+4+0+0)	P1 = 6 (1+0+4+1+0) P2 = 5 (1−2+4+1+1)
**Provider 1 (P1)** **(high volume,** **high quality)**	P1 = 4 (1−2+4+0+1) P2 = 5 (1+0+4+0+0)	P1 = 5 (1−2+4+1+1) P2 = 5 (1−2+4+1+1)
**Results for examples a and b**: dominant strategy remains to provide low quality services, but with a reduced risk of lower payment if a provider chooses to provide high quality services. A small temptation to free ride still exists.		
**5c**. Fee-For-Service Dominant Blended Payment with Bigger Incentive Payment [and lower “base” payment = fee-for-service (V_high_= 3) + capitation (1) = 4] **Using these assumptions: V_high_= 3, V_low_ =0; Q_high_=−2, Q_low_=0; C= 1; E_highQ_=1, E_lowQ_=0; I_highQ_=2, I_lowQ_=0** **Less emphasis on paying for volume of services (V_high_= 3), with small capitation rate (C= 1) and larger** **incentive payment (I_highQ_=2)**		
	**Provider 2 (P2) (“others”)** **(high volume, low quality)**	**Provider 2 (P2) (“others”)** **(high volume, high quality)**
**Provider 1 (P1)** **(high volume,** **low quality)**	P1 = 4 (3+0+1+0+0) P2 = 4 (3+0+1+0+0)	P1 = 5 (3+0+1+1+0) P2 = 5 (3−2+1+1+2)
**Provider 1 (P1)** **(high volume,** **high quality)**	P1 = 4 (3−2+1+0+2) P2 = 4 (3−0+1+0+0)	P1 = 5 (3−2+1+1+2) P2 = 5 (3−2+1+1+2)
**5d**. Capitation Dominant Blended Payment with Bigger Incentive Payment [and lower “base” payment = fee-for-service (V_high_= 1) + capitation (3) = 4] **Using these assumptions: V_high_= 1, V_low_ =0; Q_high_=−2, Q_low_=0; C= 3; E_highQ_=1, E_lowQ_=0; I_highQ_=2, I_lowQ_=0** **Less emphasis on capitation rate (C= 3), with small fee for volume of services (V_high_= 1), larger incentive** **payment (I_highQ_=2)**		
	**Provider 2 (P2) (“others”)** **(high volume, low quality)**	**Provider 2 (P2) (“others”)** **(high volume, high quality)**
**Provider 1 (P1)** **(high volume,** **low quality)**	P1 = 4 (1+0+3+0+0) P2 = 4 (1+0+3+0+0)	P1 = 5 (1+0+3+1+0) P2 = 5 (1−2+3+1+2)
**Provider 1 (P1)** **(high volume,** **high quality)**	P1 = 4 (1−2+3+0+2) P2 = 4 (1+0+3+0+0)	P1 = 5 (1−2+3+1+2) P2 = 5 (1−2+3+1+2)
Results for Examples c and d: no dominant strategy exists. Providers cannot maximize individual gain by providing low quality services. There is no incentive to free ride. If collective action is encouraged, providers will choose to maximize individual and collective rewards by delivering a high volume of high quality services.		
